# Emotional Reactivity and Regulation Relate to Surgical Treatment Decision Making Among Newly Diagnosed Women With Breast Cancer

**DOI:** 10.1002/cam4.70357

**Published:** 2024-12-09

**Authors:** Catherine Benedict, Bita Nouriani, Eric Neri, Kate Miller, Allison W. Kurian, James J. Gross, David Spiegel

**Affiliations:** ^1^ Department of Psychiatry and Behavioral Sciences Stanford University School of Medicine Stanford California USA; ^2^ Stanford Cancer Institute Stanford California USA; ^3^ Quantitative Sciences Unit Stanford University School of Medicine Stanford California USA; ^4^ Department of Medicine and of Epidemiology and Population Health Stanford University School of Medicine Stanford California USA; ^5^ Department of Psychology Stanford University Stanford California USA

**Keywords:** breast cancer, emotion regulation, emotional reactivity, treatment decision making

## Abstract

**Background:**

Despite bilateral mastectomy (BLM) for early‐stage breast cancer (BC) showing no survival benefit and increased risk compared to breast conserving surgery, some patients still choose this treatment. This study examined whether emotion reactivity and regulation influence treatment decision making among newly diagnosed women with breast cancer.

**Methods:**

Cross‐sectional survey data were analyzed as part of a larger study. Measures included the Contralateral Prophylactic Mastectomy (CPM) survey, PROMIS Anxiety scale, and Emotion Regulation Questionnaire (ERQ) Cognitive Reappraisal and Emotional Suppression subscales. Primary analysis included a logistic regression model predicting treatment choice (BLM vs. non‐BLM).

**Results:**

Participants (*N* = 137) with unilateral BC (Stages 0–III) were divided between BLM (*n* = 66) versus breast conserving surgery (i.e., non‐BLM, *n* = 71) treatment groups. Compared to the non‐BLM group, the BLM group was younger, more likely to be partnered, and had a higher household income. Women with high levels of BC‐specific worry were 3.6 times more likely to choose BLM compared to women with low levels of worry (OR = 3.09, 95% CI: 1.07–0.8.93). Those who used cognitive reappraisal were 10% less likely to choose BLM compared to women who did not use cognitive reappraisal (OR = 0.90, 95% CI: 0.82–0.99). There were no group differences in levels of generalized anxiety (OR = 0.93, 95% CI: 0.87–0.99) or emotional suppression (OR = 1.02, 95% CI: 0.90–1.16).

**Conclusions:**

Findings suggest the choice of BLM may be due, in part, to negative emotional experiences after a BC diagnosis and lesser use of reappraisal to reframe cancer‐related fears. These may be important targets of intervention to support women making BC treatment decisions.

After a breast cancer (BC) diagnosis, the choice of bilateral mastectomy (BLM) usually represents both treatment for the affected breast and prevention for the contralateral breast, with the rare exception of patients with bilateral breast tumors. Although there is no survival benefit, the rate of BLM uptake [1, 2], and specifically use of contralateral prophylactic mastectomy [3, 4, 5, 6], has been increasing in the United States for decades. Compared to more conservative treatments, BLM poses greater risks in terms of surgical complications and associated costs [7, 8, 9, 10], long‐term sequelae (e.g., numbness of the chest skin, loss of nipple sensation, chronic pain), and negative psychosocial outcomes (e.g., cosmetic concerns, worsened body image, and sexual function) [11, 12, 13, 14]. Studies describing predictors and outcomes of BLM choice have focused on patient characteristics (e.g., age, race/ethnicity), clinical factors (e.g., family history, tumor size), and other contextual factors (e.g., insurance, surgeon recommendations) [15, 16, 17, 18, 19, 20, 21]. Fewer studies have reported on the subjective experiences of women making treatment decisions.

Patients' perceptions of cancer and their emotional experiences influence BC treatment decision‐making. Fear of cancer recurrence is among the most important factors in BLM choice [22, 23]. In a sample of 550 women diagnosed with unilateral BC who elected BLM, the top reasons were to reduce the risk of cancer, enhance peace of mind, and improve survival/extend life [24]. Notably, most women reported accurate risk perceptions and acknowledged that BLM would not lead to an extension of survival [24]. In fact, quantifiable risk information have limited influence on treatment choice and improving numeric comprehension of risk probabilities has limited benefit [25, 26, 27]. Decision making is also influenced by other emotional and coping factors [27]. Higher cancer‐specific distress at baseline (pre‐surgery) predicts choice of BLM involving contralateral prophylactic mastectomy [20]. The degree to which patients are emotionally sensitive to risk probabilities also influences decision making about cancer treatment [28].

Given the important role that negative emotions play in clinical decision‐making, the use of effective emotion regulation strategies may also be crucial. Emotion regulation refers to the processes that influence which emotions are experienced, the degree to which they are experienced, and when they are experienced, and may include intentional strategies to change anticipated or actual emotional responses [29]. The Affect Regulation in Cancer (ARC) framework includes two types of emotion regulation strategies that are used across the cancer continuum, that is, cognitive reappraisal and expressive suppression [30]. Cognitive reappraisal involves changing one's appraisal of a situation in order to change their emotional response, and has been shown to be effective in downregulating negative affect [31]. In cancer situations, the use of cognitive reappraisal is associated with less psychological distress after a cancer diagnosis and during treatment [32, 33, 34]. Emotional suppression involves controlling one's outward emotional expressions so that other people are unable to recognize one's emotional state [30]. Within oncology settings, emotions and emotion regulation strategies have been shown to influence health decision‐making for both patients and health care professionals [35].

This study aimed to better understand the influence of emotional reactivity and regulation on surgical treatment decision‐making among newly diagnosed BC patients with unilateral disease. We examined associations between surgery choice (BLM vs. other breast conserving surgical options) and emotional reactivity and regulation. We hypothesized that women choosing BLM would report higher levels of BC‐specific worry and generalized anxiety and lower ability to regulate their emotions via cognitive reappraisal and expressive suppression, compared to women who chose more conservative surgery. Self‐reported reasons for choosing contralateral prophylactic mastectomy (i.e., choosing BLM) were examined and controlled for in the analyses. A better understanding of patients' emotional experiences may inform interventions to enable anxiety management through non‐surgical means.

## 1 Methods

This analysis was part of a larger study of the neurobiological and affective determinants of BLM choice and longitudinal psychosocial functioning post‐decision (NCT03050463). Only data from the baseline survey were included in the present analyses.

Participants were recruited through institutional clinical practices, social media sources, and patient advocate organizations. One hundred thirty‐seven women were enrolled (64% of the 213 potential participants that met eligibility criteria) out of a total 1187 potential participants; 33% (*n* = 395) of potential participants were from Stanford Cancer Center records, 26% (*n* = 313) from the Army of Women website, 26% (*n* = 304) from social media (e.g., Facebook), and the remaining 15% (*n* = 178) from other sources (e.g., study flyer, family or friend referral, and local BC patient organization). Participants provided informed consent. Medical variables were extracted from electronic medical records. Compensation of $250 was provided for completing baseline study activities that included a survey as well as behavioral tasks, fMRI scan, and salivary cortisol and blood sample collection. Study procedures were in accordance with ethical guidelines for human subject's research and approved by the Stanford Institutional Review Board (Protocol #34959).

### 1.1 Participants

Eligibility criteria included women diagnosed with unilateral BC (Stages 0–III) who were 18 years or older, proficient in English, and US citizens or residents. The date of study enrollment was not more than 12 months from the date of BC diagnosis, and women were eligible whether they had or had not undergone BC surgery at the time of completing the baseline survey. Due to requirements of the parent study, it was also required that women agree to suspend treatments containing benzodiazepines and steroids up to 2 weeks before and during cortisol sampling, agree to provide saliva samples and have an fMRI scan, and have no contraindications to MRI imaging. Exclusion criteria included: another active cancer within the past 10 years other than squamous cell carcinoma of the skin, pregnancy, any significant neurologic disease that could affect MRI data (e.g., dementia, Parkinson's disease, or Huntington's disease), hearing impairment, current untreated psychosis, bipolar disorder, substance/alcohol abuse/dependence, or current use of psychotropic medication 5 or more days a week.

### 1.2 Measures

Standard questionnaires collected sociodemographic and medical information. The revised version of the Contralateral Prophylactic Mastectomy (CPM) survey measured BC knowledge, risk perceptions, BC worry and concerns, and treatment decisions [24]. Two sections of the CPM survey ask about treatment decision making. The first asks respondents to rate the level of importance of different reasons for their decision about surgery (21 items) and the second asks about areas of concern related to their decision (11 items). Principal component analysis (PCAs) is a multivariate technique that can be used to reduce the dimensionality of a large dataset by summarizing the information content through a smaller set of summary indices or latent factors [36]. We conducted a PCA with varimax rotation of the treatment decision‐making items within the CPM, which revealed seven orthogonal latent factors, that is, five reasons for choosing BLM or non‐BLM surgery (i.e., cancer‐related fears, adverse medical history, cosmetic concerns, family concerns, and family cancer history) and two areas of concern about having the healthy breast removed (i.e., about the surgical procedure and about cosmetic and sexual side effects). The items that most strongly influenced each latent factor of the PCA (i.e., highest loading items) were used in analyses. Items referring to the importance of treatment decision‐making factors were dichotomized as either “extremely” or “very important” [1] versus “somewhat,” “not at all important,” or “unsure” (0); and items referring to areas of concern were dichotomized as very concerned [1] versus concerned, not concerned, or does not apply (0).

#### 1.2.1 Emotional Reactivity

Two measures were used to assess emotional reactivity. First, generalized anxiety was measured using the 4‐item PROMIS (Patient Reported Outcomes Measurement Information System)—Anxiety scale (PROMIS‐Anx), which measures the degree to which individuals felt anxiety in the past 7 days [37]. Responses are on a five‐point Likert scale from “never” (0) to “always” [5]. Scores are standardized as T‐scores with mean = 50 and standard deviation = 10, which allows results to be directly compared to the general population PROMIS‐Anx values. The PROMIS‐Anx scale is a widely used measure in oncology [38] with strong psychometric properties [39]. Second, a single item from the CPM was used as a measure of BC‐specific worry (BC‐worry), that is, “In general, how worried are you about breast cancer?” To assist interpretation, responses were dichotomized into groups reporting to be “very worried” [1] versus those who were “not at all,” “a little,” or “somewhat worried” (0).

#### 1.2.2 Emotion Regulation

The Emotion Regulation Questionnaire (ERQ) is a 10‐item scale measuring two relatively independent types of emotion regulation tendencies, represented by the Cognitive Reappraisal (6 items) and Expressive Suppression (4 items) subscales [40]. Cognitive reappraisal refers to thinking differently about a situation in order to change its meaning to then alter one's emotional experience (e.g., “When I want to feel less negative emotion, I change the way I'm thinking about the situation.”). Expressive suppression involves decreasing the outward expression of emotion (e.g., “When I am feeling negative emotions, I make sure not to express them.”) Responses are on a seven‐point Likert scale ranging from “strongly disagree” [1] to “strongly agree” [7]. Total scores were calculated with higher scores indicating a greater tendency to use the emotional regulation strategy. The ERQ has shown strong internal reliability and factorial validity in women with cancer [41] and large general community samples [42, 43].

### 1.3 Statistical Methods

Group differences between BLM and non‐BLM subgroups in sociodemographic and medical variables and CPM survey factors were evaluated using independent sample *t*‐tests and chi‐squared, and factors that were significant were included as covariates in the multivariate model predicting treatment choice. Primary analysis included a logistic regression model with treatment choice (BLM vs. non‐BLM) as the dependent variable. Predictors of treatment choice included covariates, entered in Step 1, and the primary outcomes of interest representing emotional reactivity (PROMIS‐Anx and BC‐worry) and emotion regulation strategies (ERQ Cognitive Reappraisal and Expressive Suppression subscales), entered in Step 2. Follow‐up exploratory analyses were conducted to further evaluate group differences. Effect sizes were calculated and categorized as small, medium, or large.

## 2 Results

Participants (*N* = 137) were all diagnosed with unilateral BC. Most were non‐Hispanic White (70%), partnered (69%), well educated (e.g., 77% had a 4‐year college degree or more), and had private insurance (76%).

The sample was divided between women who chose BLM (*n* = 66; 48%) as treatment for unilateral BC and those who chose a more conservative surgical approach: unilateral mastectomy or lumpectomy plus radiation (i.e., non‐BLM; *n* = 71, 52%). Women who chose BLM were younger (*p* < 0.05), more likely to be partnered/married (*p* = 0.001), and had a higher annual household income (*p* < 0.01), whereas those who chose non‐BLM were older, more likely to be single, and to have a lower household income. Women with a positive BRCA1/2 testing result were more likely to choose BLM and those that did not test their BRCA1/2 status were more likely to choose non‐BLM (*p* = 0.02). See Table 1 for sociodemographic and medical characteristics for the sample and by treatment group (BLM vs. non‐BLM).

**TABLE 1:** Sociodemographic and medical characteristics of the sample (*N* = 137).

Descriptor	Combined sample (*N* = 137)		BLM group (*n* = 66)		Non‐BLM (*n* = 71)		MWW test or chi‐squared test
	*N*	Mean (SD) or %	*N*	Mean (SD) or %	*N*	Mean (SD) or %	*p* value
Age at baseline	137	49.2 (11.2)	66	47.0 (10.1)	71	51.2 (11.7)	< 0.05
Race							
White	105	76.6%	51	77.3%	54	76.1%	0.87
Other	32	23.4%	15	22.7%	17	23.9%	
Ethnicity							
Hispanic	15	10.9%	8	12.1%	7	9.9%	0.67
Non‐Hispanic	122	89.1%	58	87.9%	64	90.1%	
Non‐Hispanic White							
No	41	29.9%	19	28.8%	22	31.0%	0.78
Yes	96	70.1%	47	71.2%	49	69.0%	
Marital status							
Single including separated, divorced, widowed, other	43	31.4%	12	18.2%	31	43.7%	< 0.01
Married or living as married	94	68.6%	54	81.8%	40	56.3%	
Number of children							
0	45	32.9%	18	27.3%	27	38.0%	0.14
1	18	13.1%	7	10.6%	11	15.5%	
2	47	34.3%	27	40.9%	20	28.2%	
3 or more	27	19.7%	14	21.2%	13	18.3%	
Education							
< 4‐year college or bachelor's degree	31	22.6%	14	21.2%	17	23.9%	0.36
4‐year college or bachelor's degree	45	32.9%	27	40.9%	18	25.4%	
> 4‐year college or bachelor's degree	61	44.5%	25	37.9%	36	50.7%	
Annual income							
< $100,000	51	38.9%	17	27.0%	34	50%	< 0.01
$100,000 or more	80	61.1%	46	73.0%	34	50%	
Staging							
DCIS	23	16.8%	11	16.7	12	16.9	0.86
Stage I	45	32.8%	23	34.8	22	31.0	
Stage II	46	33.6%	15	22.7	31	43.7	
Stage III	16	11.7%	12	18.2	4	5.6	
Stage IV	0	0.0%	0	0.0	0	0.0	
Other/Missing	7	5.1%	5	7.6	2	2.8	
Tumor Grade							
Score = 3‐5:G1 (low grade, well differentiated)	44	32.1%	20	30.3	24	33.8	0.91
Score = 6‐7:G2 (intermediate grade, moderately differentiated)	43	31.4%	24	36.4	19	26.8	
Score = 8‐9:G3 (high grade, poorly differentiated)	25	18.2%	10	15.2	15	21.1	
Missing	25	18.2%	12	18.2	13	18.3	
ER status							
Positive	109	79.6%	54	81.8	55	77.5	0.70
Negative	18	13.1%	7	10.6	11	15.5	
Unknown	10	7.3%	5	7.6	5	7.0	
PR status							
Positive	96	70.1%	45	68.2	51	71.8	0.89
Negative	29	21.2%	15	22.7	14	19.7	
Unknown	12	8.8%	6	9.1	6	8.5	
HER2/Neu status							
Positive	25	18.2%	14	21.2	11	15.5	0.44
Negative	87	63.5%	43	65.2	44	62.0	
Unknown	24	17.5%	9	13.6	15	21.1	
Missing	1	0.7%	0	0.0	1	1.4	
BRCA1/2 testing results							
Positive	9	6.6%	8	12.1	1	1.4	0.02
Negative	106	77.4%	51	77.3	55	77.5	
Not tested	21	15.3%	7	10.6	14	19.7	
Missing	1	0.7%	0	0.0	1	1.4	

Reasons for and concerns about treatment decisions were evaluated with the CPM and compared between the two groups (see Table 2). When evaluating the importance of various factors that influenced their treatment decisions, the BLM group, compared to the non‐BLM group, reported greater importance related to their desire to lower the chance of getting cancer in the healthy breast (i.e., future cancer risk, *p* < 0.001), desire to have both breasts look the same after surgery (i.e., cosmetic concerns, *p* = 0.001), considering one's partner (i.e., family concerns, *p* = 0.001), and higher levels of concern about pain at the surgical site (i.e., concerns about surgery, *p* < 0.001). No group differences were observed in the degree to which concerns about adverse medical history, family cancer history, or concern about sexual health following surgery were reasons for their surgery choice.

**TABLE 2:** Group difference between women who chose BLM versus non‐BLM surgical treatment for unilateral breast cancer.

Factors contributing to surgical decisions^a^	BLM		Non‐BLM		Area under the curve (AUC)	Success rate difference (SRD)	AUC effect size rating	Mann–Whitney *U* test, *p* value
	*N*	Median	*N*	Median				
Future cancer risk	65	1.14	66	2.00	0.13	−0.74	Large	0.000
Adverse medical history	52	4.00	50	4.00	0.48	0.03	‐none—	0.729
Cosmetic concerns	63	2.50	68	3.00	0.34	−0.33	Medium	0.001
Family concerns	65	2.50	67	2.75	0.34	−0.32	Medium	0.001
Family cancer history	56	2.50	60	2.50	0.46	−0.08	‐none—	0.439
Concerns about the surgical procedure (i.e., pain)	58	2.00	60	1.46	0.80	0.60	Large	0.000
Concerns about sexual side effects of surgery	66	1.88	69	1.75	0.52	0.04	‐none—	0.720

^a^ Factors contributing to surgical decisions were identified via principal component analysis of the contralateral prophylactic mastectomy (CPM) survey, which revealed seven latent factors representing five reasons for choosing surgery (i.e., cancer‐related fears, adverse medical history, cosmetic concerns, family concerns, and family cancer history) and two areas of concern about having the healthy breast removed (i.e., about the surgical procedure and about sexual side effects of surgery).

### 2.1 Multivariate Model Predicting Treatment Choice (BLM vs. Non‐BLM)

A stepwise logistic regression model was specified to determine the strongest predictors of surgery choice (BLM vs. non‐BLM; Table 3). Step one included a priori covariates (i.e., age, partnership status, income, and surgery time point), along with CPM survey items that were significantly associated with treatment decisions in bivariate analysis. Genetic testing results was not included as a covariate due to missing data (unknown BRCA1/2 status). The CPM survey item assessing family concerns was not included because not all participants had partners or children. To test our main hypotheses, step two included BC‐specific worry (BC‐worry), general anxiety (PROMIS‐Anx), and emotion regulation strategies of cognitive reappraisal and expressive suppression (ERQ subscales).

**TABLE 3:** Logistic regression predicting BLM surgical treatment choice.

Variable	B	SE	Wald *χ* ^2^	Pr > *χ* ^2^	OR (95% CI)
Intercept	7.30	3.35	4.75	0.03	
Age	−0.05	0.03	3.1	0.08	0.96 (0.91,1.01)
Income (> $100,000)	0.13	0.26	0.23	0.63	1.29 (0.46,3.61)
Partnership status (married)	0.91	0.29	9.65	0.002	6.18 (1.96,19.51)
Concerns about the surgical procedure	−1.10	0.35	10.04	0.002	0.11 (0.03,0.43)
Cosmetic concerns	0.54	0.26	4.21	0.04	2.94 (1.05,8.21)
Future cancer risk	2.32	0.59	15.48	< 0.0001	104.42 (10.3, > 999)
Breast cancer‐specific worry (CPM item)	0.56	0.27	4.32	0.04	3.09 (1.07,8.93)
Generalized anxiety (PROMIS anxiety T‐score)	−0.08	0.04	4.47	0.04	0.93 (0.87,0.99)
Cognitive reappraisal (ERQ subscale)	−0.10	0.05	4.52	0.03	0.90 (0.82,0.99)
Expressive suppression (ERQ subscale)	0.02	0.06	0.13	0.71	1.02 (0.90,1.16)

Consistent with our hypothesis, women with high levels of BC‐specific worry were three times more likely to choose BLM surgery compared to women with low levels of worry (OR = 3.09, 95% CI: 1.07–8.93). With respect to emotion regulation, greater use of cognitive reappraisal as an emotion regulation strategy was associated with a lower likelihood of choosing BLM; that is, a one‐point increase in ERQ cognitive reappraisal led to 10% lower likelihood of choosing BLM, whereas a five‐point increase led to a 40% lower likelihood of choosing BLM (OR = 0.90, 95% CI: 0.82–0.99). That is, women with *higher* BC‐specific worry and those who were *less* inclined to change the way they think about a situation in order to change their emotional experience were more likely to have chosen BLM, compared to women with lower BC‐specific worry and greater use of cognitive reappraisal to regulate distressing emotions. Contrary to our hypotheses, there were no differences in generalized anxiety (OR = 0.93, 95% CI: 0.87–0.99) or expressive suppression (OR = 1.02, 95% CI: 0.90–1.16) between the treatment groups.

With respect to covariates, women who reported ‘the desire to have both breasts look the same after surgery’ as an extremely or very important factor in their treatment choice were 2.94 times more likely to choose BLM compared to women that reported this was not an important factor (i.e., cosmetic concerns; OR = 2.94, 95% CI: 1.05–8.21). Those with lower concern about pain at the surgery site were also 11% more likely to choose BLM compared to women who were very concerned about pain (i.e., concerns about the surgical procedure; OR = 0.11, 95% CI: 0.03–0.43). Partnered women were 6.18 times more likely to choose BLM compared to single women (OR = 6.18, 95% CI: 1.96–19.51). Age and income did not predict treatment choice. Regarding ‘the desire to lower the chance of getting BC in the healthy breast’ as a variable, extreme data sparsity resulted in distorted OR estimates (i.e., almost all women reported this as an extremely/very important factor in their treatment choice). To check the influence of this variable, we also ran the model without it and results were consistent.

### 2.2 Post Hoc Analyses

We conducted bivariate analyses to further understand the role of BC‐specific worry and cognitive reappraisal between treatment groups (BLM vs. non‐BLM). Consistent with the multivariate model, women who chose BLM reported significantly higher levels of BC‐specific worry compared to women in the non‐BLM group (x^2^ = 6.88, *p* = 0.009), with a small effect size (SRD = 0.23, 95%CI: 0.06, 0.40). That is, 50% of women in the BLM group reported being “very worried” about BC, compared to 28% of women in the non‐BLM group. The BLM group also reported less use of cognitive reappraisal as an emotion regulation strategy, compared to the non‐BLM group (ERQ subscale, *p* = 0.056), with a small effect size (Cohen's d = 0.33). For example, 81% of women in the non‐BLM group agreed or strongly agreed with the statement, “I am very capable of controlling my emotions by changing the way I think about the situation I'm in,” whereas 63% of women in the BLM group agreed.

Exploratory analyses further examined general anxiety, given the inconsistent findings between the BC‐Worry and PROMIS‐Anx measures in the logistic regression. In bivariate analysis, there was no group difference in general anxiety (*p* = 0.58), with both groups characterized by anxiety levels that were above the general population mean (T‐score of 50) and categorized as mild anxiety. However, subgroup analyses compared women who had already completed their surgery (*n* = 47) versus those who were waiting to undergo surgery (*n* = 19) at the time of the baseline assessment in each group. Here we observed a difference between the BLM subgroups such that women who were waiting to undergo surgery reported moderate levels of anxiety (*M* = 59.9, SD = 9.3), whereas women who had completed their surgery reported mild levels of anxiety (*M* = 54.3, SD = 8.6) and a medium effect size was observed (Cohen's *d* = 0.64). A similar effect was observed for the non‐BLM group such that women who were waiting for surgery (i.e., lumpectomy or unilateral mastectomy) reported higher levels of general anxiety (*M* = 60.2, SD = 7.5) than women were had undergone their surgery before completing the baseline survey (*M* = 51.3, SD = 7.9). Findings suggest, with respect to general anxiety levels, having undergone surgery for BC led to lowered anxiety for women in both treatment groups similarly. General anxiety was moderately correlated with cognitive reappraisal (*r* = −0.45, *p* < 0.001) and weakly correlated to expressive suppression (*r* = −0.20, *p* = 0.02) and age (*r* = −0.36, *p* < 0.001), and differed by levels of BC‐specific worry (*t*[134] = −3.77, *p* < 0.001). In separate models, we examined these factors and their interaction terms with general anxiety predicting treatment choice and none of the interactions were significant predictors of treatment choice.

## 3 Discussion

In this study of newly diagnosed women with BC (Stages 0–III), we identified differences in emotional reactivity and regulation between those who chose BLM versus more conservative surgical treatment. Women with high levels of BC‐specific worry were three times more likely to choose BLM compared to women with low levels of worry. Additionally, greater use of cognitive reappraisal as an emotion regulation strategy was associated with a decrease in the likelihood of choosing BLM, controlling for BC‐specific worry, general anxiety, and other BC‐related concerns. Findings suggest the choice of BLM may be due, in part, to negative emotional experiences after a BC diagnosis and a lesser tendency or ability to reframe cancer‐related fears. These may be important targets of intervention to support women making BC treatment decisions.

Findings are consistent with prior work demonstrating that high levels of anxiety and fear after a new BC diagnosis are associated with a greater likelihood of women undergoing contralateral prophylactic mastectomy [18, 44, 45]. LeVasseur et al. reported that patients with early‐stage BC and high anxiety at initial consultation were nine times more likely to undergo aggressive surgery, including BLM for unilateral disease, compared to those with low anxiety [46]. Likewise, Rosenberg et al. reported that the main reason BC patients with unilateral disease chose BLM was to reduce anxiety about developing a second primary BC despite being aware of the low risk [24]. In our study, women in the BLM group, compared to the non‐BLM group, not only reported higher levels of BC‐specific worry, but were also more likely to report a ‘desire to lower the chance of getting cancer in the healthy breast’ as *very* or *extremely* important in their treatment decision. Although many factors influence decision making [47, 48], the current results add to the literature suggesting some women may look to surgery as a way of managing their anxiety about future cancer risk.

This study builds on prior findings by identifying emotion regulation strategies as yet another factor that influences treatment decision making. We found that greater use of cognitive reappraisal, or the ability to think differently about a situation to change its meaning and emotional impact [49], was associated with a lower likelihood of choosing BLM. Although initial appraisals of a cancer diagnosis are often experienced as fearful and distressing, reappraising the situation can be a powerful way to decrease negative emotions and refocus on other tasks at hand or alternative emotions such as hope. Cognitive reappraisal, compared to other emotion regulation strategies, is most effective when the situation is unavoidable, such as needing to make a treatment decision within a time frame [30]. Reappraisal, or cognitive reframing, is a commonly taught skill within cognitive behavioral therapy, and may be effective for supporting women as they face treatment decisions [50].

Results are also consistent with separate analyses done as part of the larger study that used fMRI to evaluate differences in neural activity between the treatment groups (BLM vs. non‐BLM) [51]. During an emotion regulation task, women were shown images and asked either to respond naturally or to try to reinterpret the meaning of the situation depicted in the picture to feel less negative (i.e., cognitive reappraisal) [51]. In response to BC‐related images, women in the non‐BLM group showed greater activation in prefrontal brain regions involved in emotion regulation than the BLM group, consistent with greater use of cognitive reappraisal [51]. Combined, the current results from patient‐reported outcomes and the previously published results of fMRI demonstrate that difficulty managing cancer‐related emotions is an important factor in treatment decision making and may lead to more aggressive surgical decisions.

In this study, BC‐specific worry, but not general anxiety, was related to surgery choice. Prior studies have found that high anxiety tends to predict more aggressive surgical decisions among women with BC [46, 52, 53]. The lack of significant findings in general anxiety was likely due to timeline differences of when the questionnaire was completed in comparison to surgery. Women who completed the questionnaire before surgery reported higher levels of anxiety than women who completed the questionnaire after surgery, irrespective of their surgery choice. Across as 12‐month period, Park et al. reported that BC surgery was a significant anxiety‐relieving factor for women; after surgery, levels of anxiety remained low until the end of adjuvant therapies, at which point anxiety levels rose again [54]. The use of surgery as a form of anxiety management poses physical, psychosocial, and financial risks. Alternatively, women may benefit from learning emotion regulation skills to manage negative emotions, which can also be applied to other cancer‐related stressors [55].

We did not find a difference in the use of emotional suppression as an emotion regulation strategy between treatment groups. Similarly, among women at high risk for BC, cognitive reappraisal, but not expressive suppression, moderated the relationship between cancer‐specific distress and decisions about chemoprevention [56]. Emotional suppression requires a conscious effort to manage the outward expression of emotions when they occur, but may result in little or no impact on the internal experience of the emotion and can lead to poorer psychological health [32, 57]. In contrast, cognitive reappraisal changes the root cause of negative emotions (i.e., the appraisal of the situation) and has been shown to be more effective than expressive suppression in downregulating negative emotions such as anxiety [58]. The effectiveness of some regulatory strategies, including expressive suppression, also varies with time such that short‐term use can be helpful, but become maladaptive in the long term [30, 59]. Future research should evaluate the use of emotion regulation strategies longitudinally and their impact as women make treatment decisions and progress through the cancer continuum.

Emotion regulation skills may be an important target for intervention to support women in feeling equipped to manage cancer‐related fears by non‐surgical means, in combination with evidence‐based strategies to support treatment decision‐making. Although the effect of cognitive reappraisal on surgery choice was small in this study, it may be that women still benefit from learning these skills to facilitate emotion regulation and decision making. Tucholka et al. reported that a decision aid provided to newly diagnosed women with BC before their first surgical consultation led to higher knowledge scores compared to a standard website, including a greater proportion of women realizing that waiting a few weeks to make a treatment decision would not affect survival [60]. Decreasing the urgency patients feel may allow women the opportunity to learn and apply emotion regulation strategies to manage their fears. Cognitive‐behavioral therapy may be used to teach women skills that enable them to reappraise, or reframe, the situation to better manage negative emotions during this time [61]. Interventions have shown benefit in reducing cancer‐related anxiety and fear of recurrence in post‐treatment survivorship and may be adapted [62, 63].

Limitations of the study include the cross‐sectional design, which precludes causal inferences. Measures of emotion regulation were limited to two types of strategies (cognitive reappraisal and expressive suppression), though women may employ other strategies (e.g., distraction, avoidance) [30]. Although we controlled for BC‐specific factors that may be relevant to treatment decision‐making, other relevant factors were not accounted for such as proximity to a healthcare facility, communication with health care professionals, or perceived support [47, 48]. Additional psychological factors such as knowledge of cancer recurrence risk, emotional sensitivity to risk information, and tolerance of uncertainty may also be important to include in future research. BRCA1/2 inherited gene mutation status was not included due to missing data. For some variables, missing data may introduce nonresponse bias. Future studies are needed to further explore these relationships and implications for clinical care and counseling.

Newly diagnosed women with BC often face difficult decisions about surgery during an emotionally charged time. Bilateral mastectomy for unilateral disease, which involves prophylactic removal of the unaffected breast, poses many risks with no survival benefit. Women who reported high levels of BC‐specific worry and less use of cognitive reappraisal as an emotion regulation strategy were more likely to choose BLM verse more conservative surgical options. Emotion regulation skills may be an important target of intervention to support women making BC treatment decisions while managing cancer‐related anxiety and fears.

## Author Contributions

**Catherine Benedict:** formal analysis (lead), writing – original draft (lead). **Bita Nouriani:** project administration (lead), writing – review and editing (supporting). **Eric Neri:** formal analysis (lead). **Kate Miller:** formal analysis (supporting). **Allison W. Kurian:** conceptualization (supporting), funding acquisition (supporting), writing – review and editing (supporting). **James J. Gross:** conceptualization (supporting), funding acquisition (supporting), writing – review and editing (supporting). **David Spiegel:** conceptualization (lead), funding acquisition (lead), investigation (lead), writing – review and editing (supporting).

## Ethics Statement

Study procedures were in accordance with ethical guidelines for human subject's research and approved by the Stanford Institutional Review Board (Protocol #34959).

## Conflicts of Interest

The authors declare no conflicts of interest.

## Data Availability

We share the neural and self‐report data on the Open Science Framework (https://osf.io/8tfps/).
